# The Role of Computed Tomography in the Management of Hospitalized Patients With COVID-19

**DOI:** 10.7759/cureus.36821

**Published:** 2023-03-28

**Authors:** Mikael Mir, Sydney Boike, Taylor Benedict, Holly Olson, Abbas B Jama, Usman Anwer, Syed Anjum Khan

**Affiliations:** 1 Medicine, University of Minnesota Medical School, Minneapolis, USA; 2 Critical Care Medicine, Mayo Clinic Mankato, Mankato, USA; 3 Radiology, Mayo Clinic Mankato, Mankato, USA; 4 Critical Care Medicine, Mayo Clinic Mankato, Minnesota, USA

**Keywords:** outcomes in covid-19 hospitalizations, computed tomography pulmonary angiography, thromboembolism, covid-19 pneumonia, ground-glass opacity, hospitalized covid 19, ct (computed tomography) imaging, covid-19

## Abstract

The emergence of SARS-CoV-2 at the end of 2019 sparked the beginning of the COVID-19 pandemic. Even though it was a novel virus, the workup of suspected COVID-19 included standard protocols used for the investigation of similar respiratory infections and pneumonia. One of the most important diagnostic tests in this regard is computed tomography (CT). CT scans have a high sensitivity in diagnosing COVID-19, and many of the characteristic imaging findings of COVID-19 are used in its diagnosis. The role of CT in COVID-19 management is expanding as more and more hospital practices adopt regular CT use in both the initial workup and continued care of COVID-19 patients. CT has helped hospitalists diagnose complications such as pulmonary embolism, subcutaneous emphysema, pneumomediastinum, pneumothoraces, and nosocomial pneumonia. Although mainly used as a diagnostic tool, the prognostic role of CT in COVID-19 patients is developing. In this review, we explore the role of CT in the management of hospitalized patients with COVID-19, specifically elucidating its use as a diagnostic and prognostic modality, as well as its ability to guide hospital decision-making regarding complex cases. We will highlight important time points when CT scans are used: the initial encounter, the time at admission, and during hospitalization.

## Introduction and background

The COVID-19 pandemic forced healthcare providers around the world into a challenging environment where it was necessary to adapt, innovate, and find creative solutions in order to provide the best patient care. Specifically, regarding imaging modalities utilized in the evaluation of respiratory illnesses, much progress had to be made as more information was unveiled regarding the novel virus.

At the start of the COVID-19 pandemic, the best methods for evaluating the new respiratory illness were unclear, and they are still debated and disputed to this day. There is no agreement amongst various governing bodies about the role of early computed tomography (CT) in COVID-19 infection. Chest CT, however, has been proven to show common imaging abnormalities that are characteristic of COVID-19 pneumonia and can be pivotal in the urgent and emergent evaluation of COVID-19 patients [1]. In comparing the use of CT to that of standard radiographic evaluation, CT can be of higher diagnostic value as it can show positive findings prior to the onset of symptoms [2]. Much of the data regarding various imaging modalities and their use in the diagnosis and management of COVID-19 is still forthcoming. However, based on clinical and diagnostic data, we feel that early use of CT is beneficial for our patients and improves outcomes.

This review will serve to analyze how the use of computed tomography has played a critical role in the way we evaluate, treat, and manage patients diagnosed with COVID-19 infection. We will begin by outlining how COVID-19 pathophysiology manifests in imaging findings on CT. We then review how CT has been used for COVID-19 evaluation in the emergency department, upon admission, and how the clinical course is monitored throughout the hospital stay.

## Review

COVID-19 findings on CT

**Figure 1:**
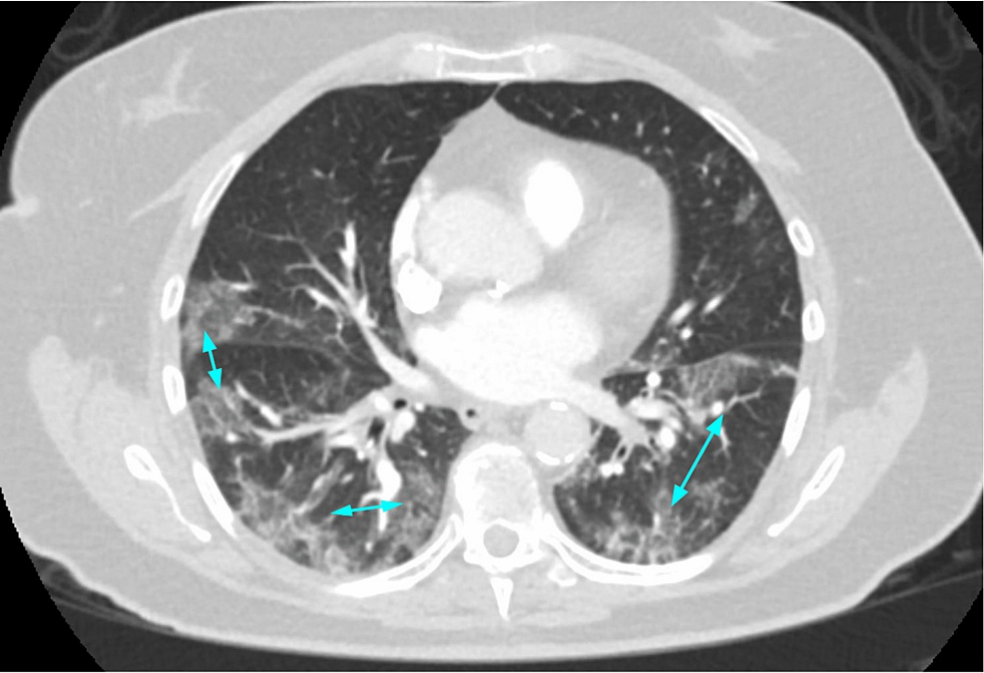
Typical peripheral, bilateral lower lung, ground-glass opacities (blue arrows) of COVID-19 pneumonia on CT.

**Figure 2:**
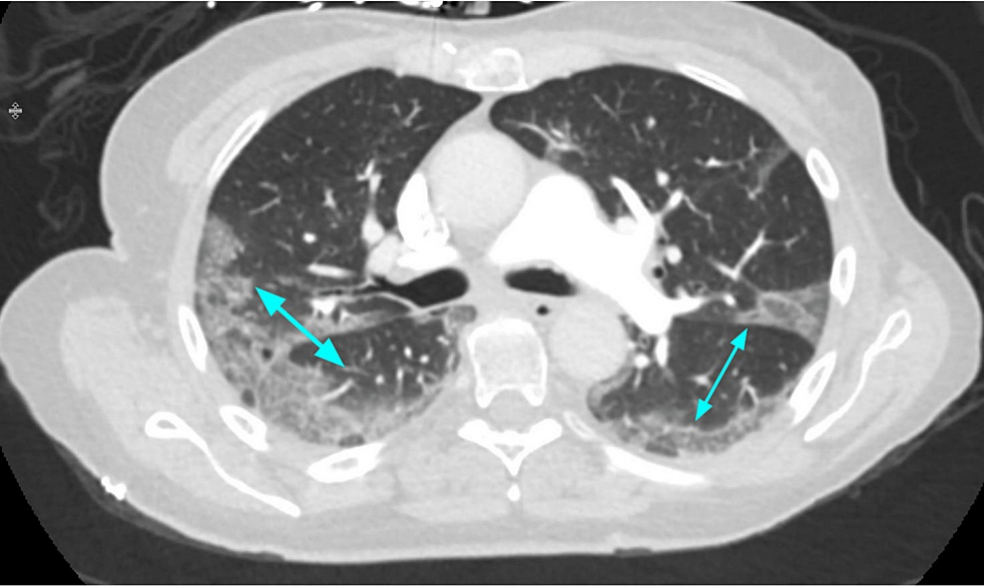
COVID-19 pneumonia with more confluent peripheral ground-glass opacities (blue arrows).

**Figure 3:**
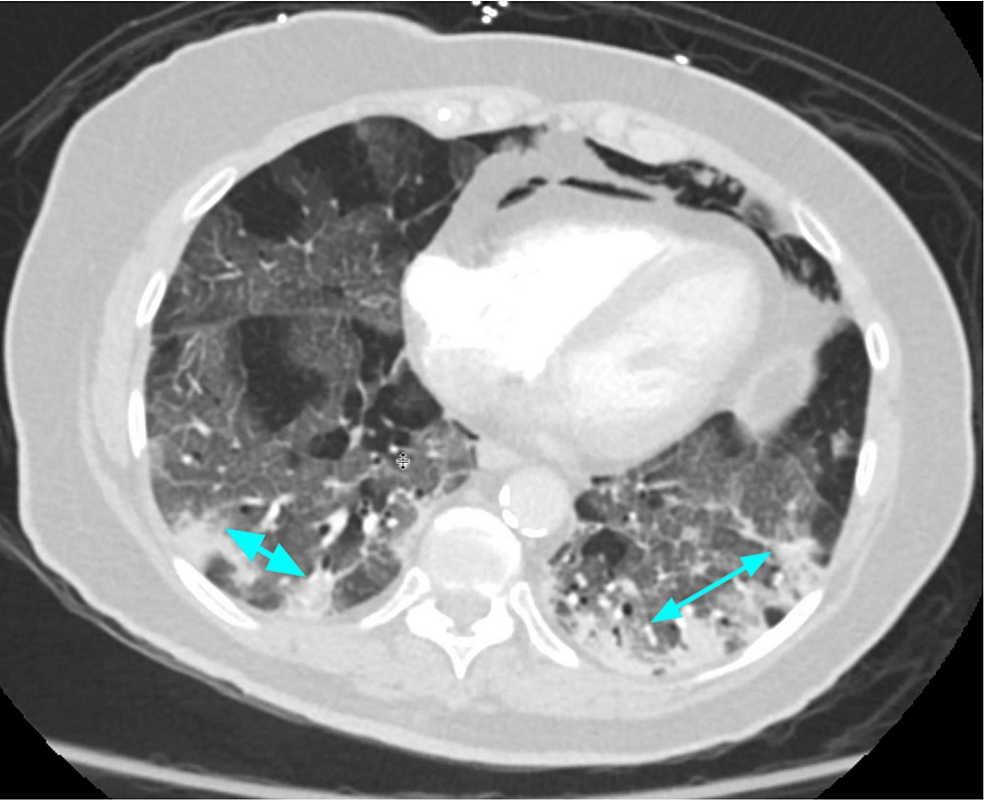
Mixed ground-glass opacities (blue arrows) and consolidation. Note the presence of pneumomediastinum as a complication of mechanical ventilation.

**Figure 4:**
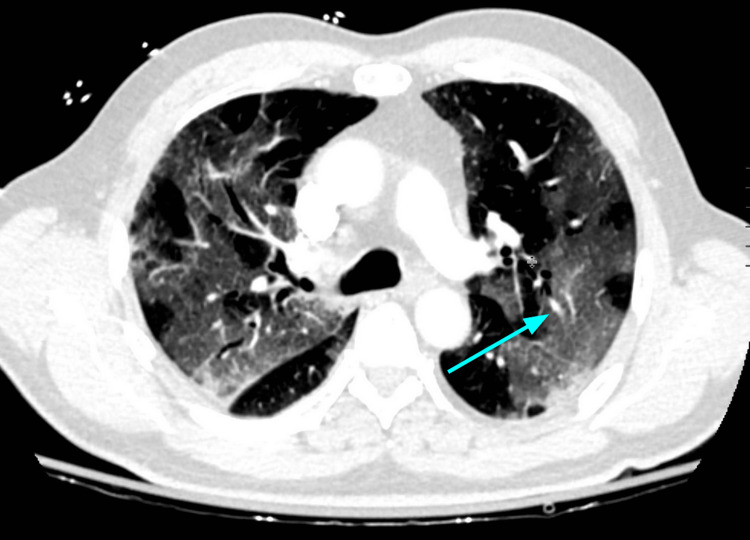
An example of subtle, crazy paving-like changes (blue arrow) in a patient with COVID-19 pneumonia, consisting of ground-glass opacities and superimposed interstitial thickening.

COVID-19’s pathophysiology helps explain its imaging appearance. The novel coronavirus that causes COVID-19 preferentially binds to angiotensin-converting enzyme 2 (ACE2) receptors, which are found in high quantities in type 2 alveolar cells. This interaction causes downregulation of the ACE2 receptors, which leads to increased capillary permeability and, eventually, interstitial inflammatory disease [3,4]. This appears on CT scans as diffuse haziness or, more commonly, as ground-glass opacities (GGOs) (Figures 1-2). As more virus binds to ACE2 receptors, the alveolar cells begin to undergo apoptosis, which is seen on CT as consolidations [3,5]. Multifocal bilateral patchy GGOs and consolidation (Figure 3) are the most common radiological findings seen in chest CT [6]. GGOs are most prominent in the peripheral lower lung zone [7]. Another common finding is the "crazy paving sign" that indicates interlobar and intralobular septal thickening [4] (Figure 4). Bronchiectasis and pleural thickening are less common findings seen in the later stages of the disease [2].

In a retrospective study of 21 patients with confirmed positive COVID-19 by Pan et al., it was found that there were four major stages of CT findings related to COVID-19 [8]. Stage 1 (0-4 days) tends to show GGOs (75%), stage 2 (5-8 days) shows an increase in crazy paving pattern (53%), stage 3 (9-13 days) shows consolidation (91%), and stage 4 (greater than or equal to 14 days) shows gradual resolution of consolidation (75%) without crazy-paving pattern [8]. These findings are ultimately not unique to COVID-19, and thus a CT cannot be relied on solely to diagnose COVID-19. However, along with molecular testing, clinical signs, and symptoms, it can be used as a rapid tool to aid clinicians in determining COVID-19 probability and disease severity [9].

CT on initial encounter

The COVID-19 pandemic flooded emergency departments with infected patients seeking care. Although laboratory tests were developed to detect the virus, given the delay in getting results and less-than-ideal sensitivity, CT was often obtained at presentation to aid in supporting the diagnosis and assessing disease severity. 

The American College of Radiology initially did not recommend CT as a first-line diagnostic test for COVID-19 pneumonia [10]. Although highly sensitive to COVID-19, specificity was felt to be lacking, and CT had additional downsides that made it challenging to use. For example, after a suspected COVID-19 patient was scanned, the CT scanner had extended downtime due to the need to properly sanitize the scanner and CT room before the next patient entered [11]. Additionally, there were increased staff exposures with transport and placement in a CT scanner, and not every hospital had the resources to scan every potentially unstable suspected COVID-19 patient in the emergency department [11]. As more efficient procedures were developed, CT use became more frequent in the initial encounter. Due to pulmonary involvement by the virus, some have strongly recommended chest CT for both initial and follow-up evaluation for moderate-severe cases [2]. Additionally, in the early stages of COVID-19 infection, CT imaging can be of higher diagnostic value than chest radiography since CT findings may be present before symptom onset [2]. 

Reverse transcription polymerase chain reaction (RT-PCR) screening tests are generally the first line in the detection of COVID-19 infection. However, these tests have been noted to have variable sensitivity. One study found that during the first week after symptom onset, false negative rates ranged from 2% to 29% [12]. In another study, patients with negative RT-PCR results with CT findings indicative of COVID-19 eventually tested positive with serial sampling [13]. CT has thus proven to be helpful for supporting a diagnosis in clinically likely cases with an initial false-negative RT-PCR test. It has also been shown to correlate positively with lab-confirmed positive COVID-19 infection [2]. A study of 51 patients early in the pandemic actually showed that the sensitivity of chest CT was greater than RT-PCR by 98% vs. 71%, respectively [14]. 

While RT-PCR testing can be used to determine the presence of COVID-19, unlike CT, it cannot determine disease severity [15]. Disease severity can be graded on CT at the initial presentation with a 25-point CT severity score. In a study of 902 patients, it was found that patients’ CT severity scores at initial presentation positively correlated with inflammatory lab markers, length of hospital stays, and oxygen requirements for those infected with COVID-19 [16]. This further supports the idea that CT cannot only provide information regarding disease presence and severity but also potentially predict clinical course and prognosis.

CT on admission

Obtaining a baseline CT on admission has increasingly become common practice in the hospital management of COVID-19 patients to monitor disease progression and provide a semi-quantitative measurement of outcomes. Ahlstrand et al. produced a visual scoring system to measure COVID-19 severity on hospital admission [17]. This was determined by radiologists reading CT scans that used a standardized protocol to give the severity score as well as temporal development of GGO to consolidation. They found that in patients less than 70 years old, the severity score on admission closely correlated with length of stay [17]. More extensive tissue damage on CT at the time of admission was scored higher, and the higher the score, the longer the hospital stay. Additional factors used to predict the length of stay that were of statistical significance included the patient’s age and CRP level at admission [17]. Another group had similar findings, with more severe lung tissue damage at admission being a predictor of progression to more severe pneumonia [18]. These studies show that at the time of admission, patients with more severe lung damage were more likely to have a longer stay in the hospital. As for predicting ICU admission, Khosravi et al. showed that CT severity scores were predictive of ICU admission, intubation, and mortality in COVID-19-positive patients and that those with severity scores above a certain threshold were more likely to need ICU admission. Additionally, reticular patterns on CT indicated a lower likelihood of ICU admission and mortality [18]. While these studies were based on the qualitative assessment of CT scans by radiologists, other studies have explored the use of AI technology to output a quantitative severity score. Fang et al. found that their AI-based severity scoring method had a stronger correlation with length of stay than that of radiologists [19]. The ability to use CT as a predictive marker allows clinicians to estimate prognosis, admit patients to the ICU earlier, and predict possible complications, such as cytokine storms and lymphopenia [4]. In a pandemic, when hospital capacity and resources are limited, it is advantageous to use such a tool to anticipate the needs of the patient for more efficient hospital placement.

CT during hospitalization

During hospital admission, CT can directly visualize lung pathology, help guide treatment, monitor disease progression, and identify harmful sequelae of COVID-19 pneumonia. CT scans of patients with COVID-19 pneumonia can identify multiple additional comorbidities such as pleural effusion, pericardial effusion, emphysema, bronchiectasis, fibrosis, pneumothorax, cardiomegaly, atelectasis, and mediastinal lymphadenopathy [18]. Treatment during hospital admission can then be tailored to CT findings. For example, antibiotics can be initiated for early signs of bacterial pneumonia or empyema on CT. Vigorous pulmonary hygiene can be performed in response to findings of atelectasis. Furthermore, robust diuresis can be started for signs of pulmonary edema, early prone position changes for dependent atelectasis, and medical therapies for pulmonary emboli or organizing pneumonia.

**Figure 5:**
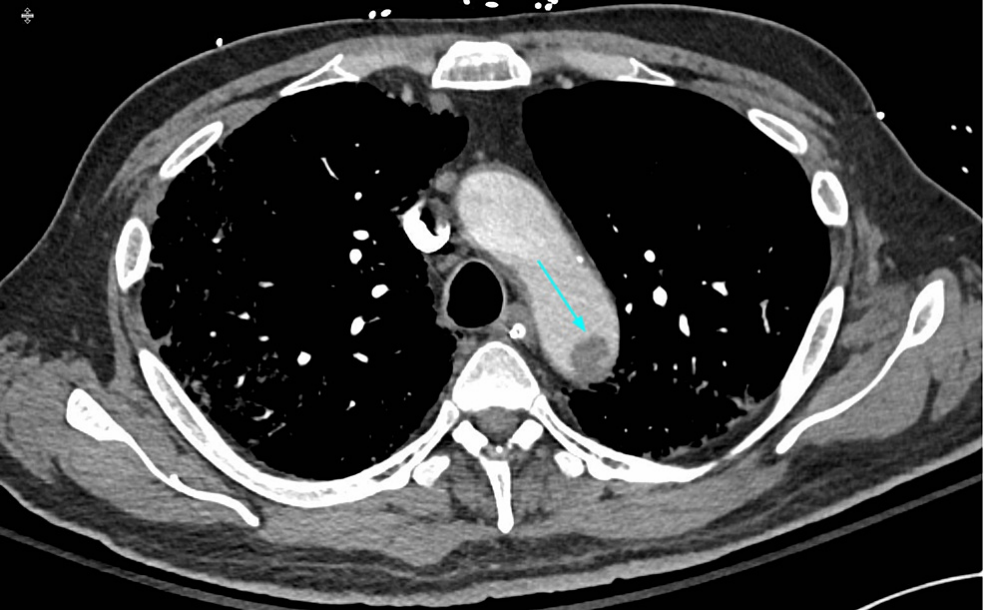
Aortic arch thrombus (blue arrow) in a patient with COVID-19 pneumonia.

**Figure 6:**
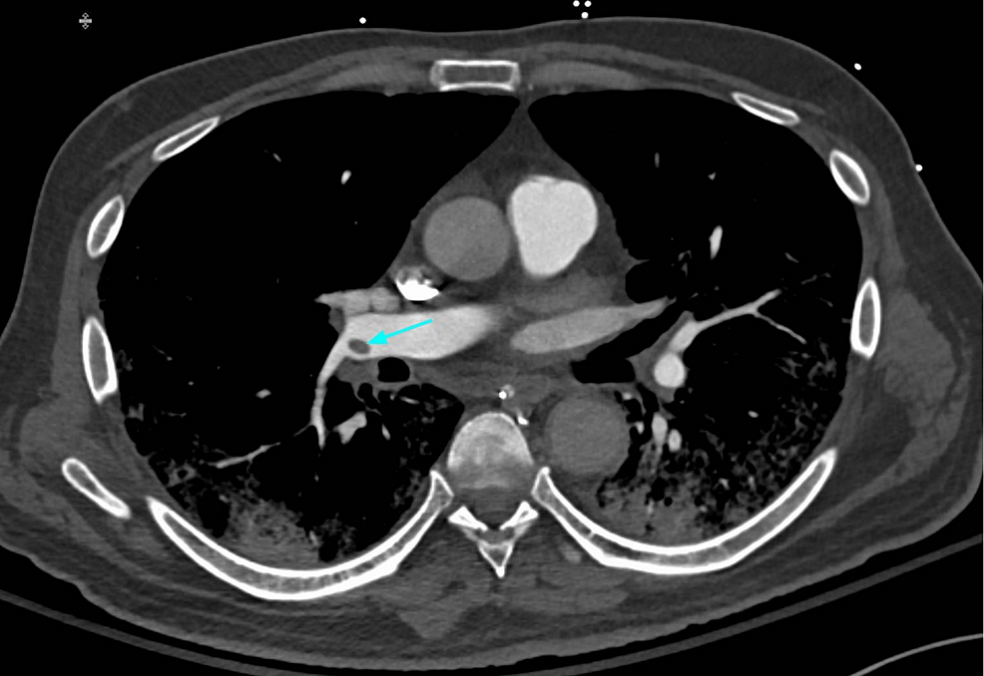
The same patient with a right distal main pulmonary artery embolus (blue arrow). Peripheral pulmonary involvement with COVID-19 pneumonia is shown.

**Figure 7:**
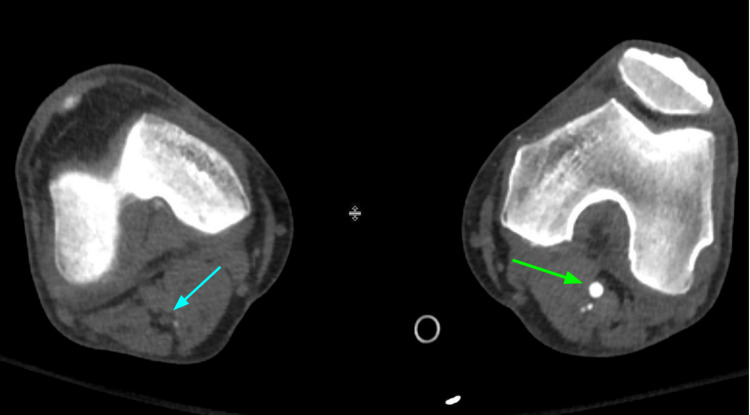
The same patient with popliteal arterial thrombosis on the right. Non-enhancement of the right popliteal artery (blue arrow) when compared to the left in this lower extremity (green arrow) CT angiogram is shown.

Hospitalized COVID-19 patients are predisposed to higher rates of thromboembolism due to high levels of systemic inflammation, immobilization, and diffuse intravascular coagulation. CT pulmonary angiography (CTPA) can play an important role in the diagnosis and management of acute pulmonary embolism and other thromboembolic events (Figures 5-7). Adverse changes in coagulation are often seen secondary to COVID-19. A meta-analysis of 1105 patients by Xiong et al. showed that prothrombin time and D-dimer values were significantly higher in patients with severe COVID-19 as compared to patients with a milder form of the disease [20]. Additionally, increased levels of D-dimer can be used as a strong predictor of mortality during hospitalization [21]. This alteration in coagulative physiology predisposes hospitalized COVID-19 patients to higher rates of thromboembolic incidents. A study by Klok et al. during the early stages of the pandemic found that the incidence of thrombotic complications for ICU patients with COVID-19 infections was 31% [22]. The prevalence of acute pulmonary embolism identified by CTPA ranges from 14-30% [23]. The National Institute for Public Health of the Netherlands recommends CTPA be reserved for COVID-19 patients with significantly elevated D-dimer (2000-4000 μg/ml) on admission or those with increasingly trending values during hospitalization [24]. Given the high risks of thromboembolic events in hospitalized COVID-19 patients, CTPA can play an important diagnostic role and help guide treatment for complex cases.

In addition to severity scores during the initial encounter or at the time of admission, chest CT severity scores during hospital admission can be used to provide prognostic information on hospital outcomes as well. A retrospective cohort study on 262 hospitalized COVID-19 patients by Abbasi et al. found that mortality was significantly higher in patients with higher CT severity scores even after adjusting for confounding variables such as clinical data, demographics, and laboratory values [11]. Li et al. explored a modified CT scoring system to assess its efficacy for determining disease severity and predictive value. The researchers classified the extent of certain common CT findings (GGOs, consolidation) in all zones of the lung using a Likert scale ranging from 0 to 4 [25]. They found that severely and critically ill patients had higher scores for GGOs, consolidation, crazy-paving pattern, and overall lung involvement as compared to patients with moderate severity. The GGO score in the second week of hospital admission had a practical predictive value for evaluating the escalation of disease severity during hospitalization for moderately severe patients. Finally, the effectiveness of the scoring system dropped during the recovery stage of the disease, and they recommended that the use of CT scans for this purpose be reduced once the patient progressed to this stage. CT scores at the time of admission and during hospitalization can both provide prognostic information that is valuable to clinicians and patients.

Late CT scan

In our review of the literature, we noticed that abnormal CT findings could remain present for some time. We summarize the abnormalities, possible discerning factors, and durations mentioned in the literature.

One single-center prospective cohort study evaluating the 90-day outcome of hospitalized patients with severe COVID-19 found that about 60% of patients had residual abnormalities at the end of the 90 days [26]. CT abnormalities included GGO (100%), reticular interstitial pattern (73%), crazy paving appearance (55%), air space consolidation (65%), bronchial wall thickening (33%), bilateral involvement (88%), five lobe involvement (73%), and peripheral distribution (78%) [26]. Shi et al. found that the initial CT showed GGO, but after several weeks, the predominant findings (53%) were consolidations and mixed pattern CT [27]. Nalbandian et al., in their literature review, describe the CT findings of three studies [28] as follows: one study by Huang et al. noted that 50% of 349 patients had evidence of at least one abnormal CT finding six months after COVID-19, and the majority were GGOs [28,29]. The other two studies by Zhao et al. and Shah et al. noted fibrotic changes three months after COVID-19 in 25% and 65% of patients, respectively [30,31]. Studies by Han et al. and Liu et al. noted that the residual abnormalities on CT findings tended to display a "tinted sign" or "melting sugar sign," which they defined as the gradual extension and consolidation of the GGO and enhanced attenuation in the image [32,33]. Liu et al. further stated that these signs imply a continuing resolution of the lung findings [33]. There are a few case reports and case series that report long-term complications of atelectasis, pleural effusions, occlusion of common femoral arteries, saddle pulmonary embolism, cysts, cavitation with sequelae of superinfection from Aspergillus fumigatus, pneumothorax, and interstitial fibrosis, in addition to the GGOs reported in previous studies [34-43].

A case series of 143 patients published in Rome found that COVID-19 symptoms persisted for 60 days [44]. The most common symptoms reported were fatigue (53.1%) and dyspnea (43.4%) [44]. Following patients for six months after a COVID-19 diagnosis, Huang et al. also found fatigue in 63% of the patients, among other symptoms such as depression, anxiety, and sleep difficulties [29]. They also noted that those who developed serious complications and abnormal imaging in their study were among those who were severely ill in the hospital [29].

These persistent symptoms can be a clue to an underlying pathologic process. Myall et al. used this in discriminating which patients to screen [45]. Their study attempted to elucidate the incidence of progression of inflammatory interstitial lung disease (ILD) that patients might experience following a bout of COVID-19. Their findings noted that 39% had persistent symptoms, and roughly 5% developed ILD and substantial functional deficits [45]. In a study by Han et al., 35% (40/114) of patients had fibrotic changes at six months, which were noted in older patients with prolonged lengths of hospital stay [32]. The greatest prognostic factors associated with whether patients developed fibrotic changes included ARDS, an initial CT imaging score ≥ 18, and the frequency of non-invasive mechanical ventilation [32]. Abdel-Hamid et al. found male gender, high BMI, an initial chest CT of consolidation or mixed consolidation with GGOs, lymphocytopenia, elevated serum CRP, and elevated ferritin were the most significant predictors of pulmonary residuals [46].

## Conclusions

The use of computed tomography in the evaluation of COVID-19 patients is multifaceted and extensive, as discussed in this review. CT has proven to be useful in the emergency department setting and in those who have been admitted and require serial imaging to monitor progression or lack thereof. Notably, CT is useful in early evaluation, as abnormal findings can be seen on it prior to the onset of symptoms. Additionally, CT has been shown to confirm COVID-19 infection in clinically likely cases with a false negative PCR test, a quality that makes it particularly useful in the emergency department where appropriate quarantine procedures are a priority. Regarding patient admission to the hospital, current knowledge of common COVID-19 findings is useful in predicting the length of stay and the need for ICU admission and thus can help in coordinating care plans and can positively impact patient outcomes. With longer patient hospitalizations, CT can be utilized to define other pathologies, such as pulmonary embolism, pleural effusion, and pneumothorax. It can be utilized to monitor COVID-19 disease progression or regression, thus guiding patient management. And finally, regarding CT use in hospitalized patients, CT severity scores on hospital admission have a useful role in determining the prognosis for patients moving forward. The use of CT in the evaluation of COVID-19 infection has been shown to be helpful at all stages of care, emergent or non-emergent, inpatient or outpatient. We believe it offers tremendous benefits for the evaluation and management of COVID-19 patients and should be utilized to its full potential for reducing morbidity and improving patient outcomes.
